# MEK5-ERK5 Axis Promotes Self-renewal and Tumorigenicity of Glioma Stem Cells

**DOI:** 10.1158/2767-9764.CRC-22-0243

**Published:** 2023-01-30

**Authors:** Kazuya Fukasawa, Jiajun Lyu, Takuya Kubo, Yuki Tanaka, Akane Suzuki, Tetsuhiro Horie, Akane Tomizawa, Ryoma Osumi, Sayuki Iwahashi, Kazuya Tokumura, Misato Murata, Masaki Kobayashi, Tomoki Todo, Atsushi Hirao, Eiichi Hinoi

**Affiliations:** 1 Laboratory of Pharmacology, Department of Bioactive Molecules, Gifu Pharmaceutical University, Gifu, Japan.; 2 Medical Research Institute, Kanazawa Medical University, Ishikawa, Japan.; 3 Division of Innovative Cancer Therapy, Institute of Medical Science, The University of Tokyo, Tokyo, Japan.; 4 WPI Nano Life Science Institute (WPI–Nano LSI), Kanazawa University, Kanazawa, Ishikawa, Japan.; 5 Cancer and Stem Cell Research Program, Division of Molecular Genetics, Cancer Research Institute, Kanazawa University, Kanazawa, Ishikawa, Japan.; 6 United Graduate School of Drug Discovery and Medical Information Sciences, Gifu University, Gifu, Japan.

## Abstract

Glioma stem cells (GSC) promote the malignancy of glioblastoma (GBM), the most lethal brain tumor. ERK5 belongs to the MAPK family. Here, we demonstrated that MAPK kinase 5 (MEK5)-ERK5-STAT3 pathway plays an essential role in maintaining GSC stemness and tumorigenicity by integrating genetic and pharmacologic manipulation and RNA sequencing analysis of clinical specimens. ERK5 was highly expressed and activated in GSCs. *ERK5* silencing by short hairpin RNA in GSCs suppressed the self-renewal potential and GBM malignant growth concomitant with downregulation of STAT3 phosphorylation. Conversely, the activation of the MEK5-ERK5 pathway by introducing *ERK5* or *MEK5* resulted in increased GSC stemness. The introduction of STAT3 counteracted the GSC phenotypes by *ERK5* silencing. Moreover, ERK5 expression and signaling are associated with poor prognosis in patients with GBM with high stem cell properties. Finally, pharmacologic inhibition of ERK5 significantly inhibited GSC self-renewal and GBM growth. Collectively, these findings uncover a crucial role of the MEK5-ERK5-STAT3 pathway in maintaining GSC phenotypes and GBM malignant growth, thereby providing a potential target for GSC-directed therapy.

**Significance:**

In this study, we demonstrated that MEK5-ERK5-STAT3 axis plays a critical role in maintaining stemness and tumorigenicity in GSCs by using genetic, pharmacologic, and bioinformatics tools, identifying the MEK5-ERK5-STAT3 axis as a potential target for GSC-directed therapy.

## Introduction

Glioblastoma (GBM) is the most malignant and therapeutically intractable primary brain tumor and it accounts for more than 45% of malignant brain tumors (1). GBM contains a subpopulation of cells, glioma stem cells (GSC), which are at the hierarchal apex of differentiation status (2). GSCs display extensive self-renewal and multilineage differentiation. GSCs are strongly associated with enhanced tumorigenesis, metastasis, recurrence, and resistance to treatment (3). Recent studies also reported that GSCs promote cancer invasion, immune evasion, tumor angiogenesis, and the recruitment of tumor-associated macrophages (4). These findings underscore the need to identify GBM novel targets, particularly for GSCs, to improve therapeutic intervention.

MAPK is a key signaling element that controls cellular processes such as proliferation, differentiation, apoptosis, and stress responses (5). The MAPK family consists of ERK 1 and 2 (ERK1/2), ERK5, c-Jun amino-terminal kinase, and p38 (6). Considering that dysregulation or inadequate functioning of MAPKs is associated with cancer initiation and progression (5), MAPKs are regarded as promising therapeutic targets. ERK5 is a relatively recently identified MAPK (6). Upon activation, ERK5 directly phosphorylates various downstream targets such as CREB, CX43, KLF2/4, p90RSK, and MEF2C, and plays essential roles in multiple fundamental cellular functions including proliferation, differentiation, migration, and survival (7). We recently demonstrated that ERK5 directly phosphorylates SMAD-specific E3 ubiquitin protein ligase 2 (SMURF2) to maintain the stemness of mesenchymal stem cells, contributing to bone homeostasis (8, 9). As ERK5 has a relatively large carboxy-terminal domain with a unique structure not conserved in other MAPKs, it can be autophosphorylated and exerts functions different from those of other MAPKs (7).

MAPK/ERK kinase 5 (MEK5; MAP2K5) is the only MEK to directly activate ERK5 through specific phosphorylation of the Thr218 and Thr220 residues of the Thr-Glu-Tyr (TEY) motif in the N-terminal activation loop (10). The MEK5-ERK5 axis is driven by various factors such as hypoxic conditions, oxidative stress, proinflammatory cytokines, and mitogens (11). Previous studies suggested that the MEK5-ERK5 axis is implicated in inflammation and cardiovascular diseases, such as atherosclerosis (12, 13). Furthermore, dysregulated the MEK5-ERK5 axis is associated with tumor growth, invasion, metastasis, poor prognosis, and therapeutic resistance in various tumor types such as breast cancer, prostate cancer, colon cancer, and lung cancer (14–19).

Moreover, recent studies revealed that pharmacologic and genetic inhibition of ERK5 signaling compromises cancer stem cell (CSC) phenotypes in leukemia and colon cancer (20, 21), although the effects of ERK5 inhibition were not tested *in vivo*. These reports raise the possibility that ERK5 also plays a very important role in maintaining the stemness of GSCs. In addition, the functional role of the MEK5-ERK5 axis in GSCs stemness regulation and tumorigenesis remains unknown *in vitro* and *in vivo*, regardless of previous reports showing that ERK5 inhibition in differentiated glioma cells decreases tumor cell growth and reduces drug resistance (22).

Here, we aimed to determine whether MEK5-ERK5 axis in GSCs could be a novel therapeutic target by integrating genetic and pharmacological manipulation in GSCs and RNA sequencing (RNA-seq) analysis of clinical specimens.

## Materials and Methods

### Cell Culture

HEK293T cells and HEK293GP cells were purchased from the RIKEN Cell Bank and Takara Bio, respectively. These cells were cultured at 37°C in a 5% CO_2_ incubator and maintained in DMEM supplemented with FBS. Human patient-derived GBM cell lines TGS-01 and TGS-04 were established as described previously (23). The use of these human materials and protocols were approved by the Ethics Committees of Gifu Pharmaceutical University (Gifu, Japan) and the University of Tokyo (Tokyo, Japan). These cells were confirmed as GSCs and cultured in neurosphere medium containing DMEM/F12 (FUJIFILM Wako Pure Chemical) supplemented with recombinant human EGF at 20 ng/mL (FUJIFILM Wako Pure Chemical), recombinant human basic FGF at 20 ng/mL (FUJIFILM Wako Pure Chemical), B27 supplement without vitamin A (Gibco), and GlutaMAX (Gibco).

### Orthotopic Xenograft Model of GSC-derived GBM and Histology

An orthotopic xenograft model of GSC-derived GBM was generated by transplantation of 5 ×  10^4^ TGS-01 GSCs into the brain of 4-week-old female nude mice (BALB/cSlc-nu/nu, SLC). Briefly, a small burr hole was drilled in the skull, 0.5 mm anterior and 2.0 mm lateral to the bregma with a microdrill, and dissociated cells were transplanted at a depth of 3 mm below the dura mater. For XMD8-92 pretreatment experiment, TGS-01 GSCs were treated with 5.0 μmol/L XMD8-92 for 24 hours, and XMD8-92 was subsequently washed off; 5 ×  10^4^ dissociated cells were transplanted into the brain of nude mice following XMD8-92 removal. Mice were sacrificed at the indicated timepoints or upon occurrence of neurologic symptoms. Mouse brains were fixed in a 4% paraformaldehyde solution, embedded in paraffin, and sectioned at a thickness of 5 μmol/L. Sections were stained with hematoxylin and eosin (H&E) and captured using a BZ-X810 fluorescence microscope (Keyence). The tumor area in each section was outlined and calculated using NIH ImageJ. Slide tumor volumes were calculated by multiplying tumor area with slice thickness of 5 μmol/L, and brain tumor volumes were approximated by summation of the slide tumor volumes for each animal. All animal experiments were approved by the Committees on Animal Experimentation of Gifu Pharmaceutical University (Gifu, Japan) and were performed in accordance with the guidelines for the care and use of laboratory animals. The number of animals used per experiment is stated in the figure legends.

### Plasmids

Plasmids pLKO.1 puro (#8453, deposited by Bob Weinberg) and pMXs-Stat3-C (#13373, deposited by Shinya Yamanaka) were obtained from Addgene. pLKO.1.sh*ERK5*-1 (#0000232396) and pLKO.1.sh*ERK5*-2 (#0000010275) were purchased from Sigma-Aldrich. pMX-*ERK5* and pMX-*DN-ERK5* plasmids were generated by subcloning into pMX vector from pcDNA3-Erk5(WT) and pcDNA3-Erk5AEF vectors, respectively, which were generously provided by Dr Jiing-Dwan Lee (Scripps Research Institute, San Diego, CA).

### Retroviral and Lentiviral Transfection

Vectors were transfected into HEK293GP cells or HEK293T cells using the calcium phosphate method. Virus supernatants were collected 48 hours after transfection and then cells were infected with virus supernatants for 24 hours in the presence of 2 μg/mL polybrene. Cells were then subjected to selection by culture with 1 μg/mL puromycin for 3 days before usage for experiments.

### Tumor Sphere Formation Assay and *In Vitro* Limiting Dilution Assay

For sphere formation assay, cells were dissociated into single cells with StemPro Accutase (Gibco). Cells were then plated in 96-well Costar ultra-low attachment plates at 1  ×  10^3^ cells per well with neurosphere medium mixed with 1.0% methylcellulose. Tumorsphere number and size were measured on day 7. For *in vitro* limiting dilution assays, cells were plated in 96-well plate at 1, 5, 10, 20, 40, or 80 cells per well, with 10 replicates for each cell number. The presence of tumorspheres in each well was examined on day 7. Limiting dilution assay analysis was performed using online software (http://bioinf.wehi.edu.au/software/elda/). Sphere formation was estimated by scoring the number of spheres larger than 50 μm.

### Cell Viability Assay, Apoptosis Assay, and Migration Assay

Cells were dissociated into single cells with StemPro Accutase (Gibco). Cell number was then evaluated by trypan blue dye exclusion assay (0.04% in PBS) using an optical microscopy. Apoptosis assay was conducted using PE-Annexin V (BD Biosciences) and 7-Amino-Actinomycin D (BD Biosciences) by flow cytometry on Beckman CytoFLEX S (Beckman Coulter). The migration ability was evaluated by a wound healing assay. Cells were seeded and incubated until they reached 90%–100% confluence. A 100 μL pipette tip was used to make cross lines, and the debris was washed away with PBS. The areas of the wounds were imaged with an optical microscope at 0 and 24 hours and analyzed using ImageJ software (NIH).

### Immunoblotting Analysis

Cultured cells were solubilized in lysis buffer (10 mmol/L Tris-HCl, 150 mmol/L NaCl, 0.5 mmol/L ethylenediaminetetraacetic acid (EDTA), 10 mmol/L NaF, 1% Nonidet P-40, pH 7.4) containing protease inhibitor cocktail. Samples were then subjected to SDS-PAGE, followed by transfer to polyvinylidene difluoride membranes and subsequent immunoblotting assay. The primary antibodies used were: anti-ERK5 (1:1,000, #3371), anti-c-Myc (1:1,000, #5605), anti-Sox2 (1:1,000, #14962), anti-MEK5 (1:1,000, #91670), anti-Stat3 (1:1,000, #9132) and anti-phospho-Stat3 (Y705; 1:1,000, #9145) and (all from Cell Signaling Technology); anti-β-Actin (1:2,000, #4778, Santa Cruz Biotechnology, Inc.); and anti-LaminB1 (1:2,000, #MABS492, EMD Millipore). Primary antibodies were diluted with blocking solution (5% skim milk). Quantification was performed by densitometry using ImageJ.

### Real-time Quantitative PCR

Total RNA was extracted from cells, followed by synthesis of cDNA with reverse transcriptase and oligo-dT primer. The cDNA samples were then used as templates for real-time PCR analysis, which was performed on an MX3005P instrument (Agilent Technologies), by using specific primers for each gene (Supplementary Table). Expression levels of the genes examined were normalized by using the *GAPDH* expression levels as an internal control for each sample.

### Single-cell RNA-seq Data Analysis

We obtained the expression and annotation data (GSE84465) from Gene Expression Omnibus and analyzed them using “Seurat” package on R (ver. 4.0.2; ref. 24). For quality control, cells expressing less than 600 genes, more than 6,000 genes, or more than 100,000 counts were extracted in advance. Next, we performed sctransform normalization with default settings, principal component analysis (PCA), and uniform manifold approximation and projection. Then, we clustered the data and defined *EGFR*^+^ cells as GBM cells, as per a previous study (24).

We also analyzed another single-cell RNA-seq (scRNA-seq) data reported in 2021 (25). The expression data were normalized by “NormalizeData” function in Seurat. Next, data scaling, PCA, and clustering were performed.

Single-sample gene set enrichment analysis (ssGSEA) and GSEA were performed using “GSVA” and “clusterProfiler” packages, respectively. Wilcoxon rank-sum test was performed for differential expression analysis using “presto” package.

### Bulk RNA-seq Data Analysis

For comparing *ERK5* expression between normal and GBM tissues, we analyzed the previous reported data (26). We also analyzed the data of GBM tissues (e.g., grade, *MGMT* methylation status) in The Cancer Genome Atlas (TCGA), the Chinese Glioma Genome Atlas (CGGA), and Rembrandt databases. Statistical significance was determined using Wilcoxon rank-sum test followed by Bonferroni correction. For correlation analysis, we calculated Pearson correlation coefficient.

### Survival Analysis

Clinical data of patients with GBM were obtained from the CGGA database. Survival analysis was performed with log-rank test using survival package, and Kaplan–Meier curves were plotted by “survminer” package.

### Statistical Analysis

Unless otherwise specified, Student *t* test and one-way ANOVA with *post hoc* Bonferroni test were used to calculate statistical significance. Throughout this study, *P* < 0.05 was considered statistically significant.

### Data Availability

The bioinformatics data used in this study are openly available in Gene Expression Omnibus (https://www.ncbi.nlm.nih.gov/geo/), GlioVis (http://gliovis.bioinfo.cnio.es/), the Broad Institute Single-Cell Portal (https://singlecell.broadinstitute.org/single_cell/study/SCP503), and the CGGA (http://www.cgga.org.cn/) databases.

## Results

### ERK5 is Associated with Stem Cell Phenotypes in GSCs

**FIGURE 1:**
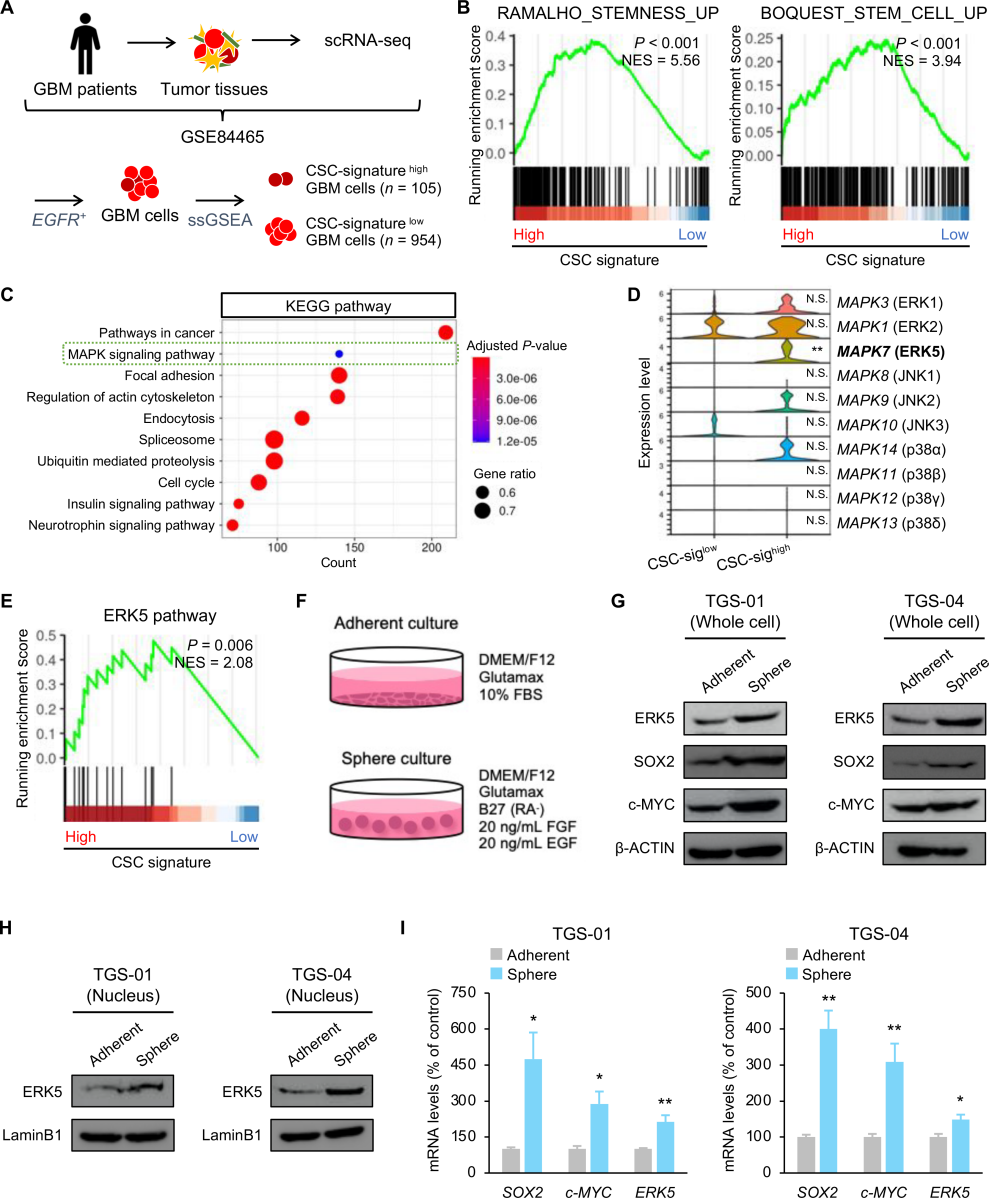
ERK5 is associated with stem cell properties in GSCs. **A,** Experimental schemes for scRNA-seq of GBM cells. **B,** GSEA of stemness-related and stem cell–related gene sets in CSC-signature^high^ GBM cells. **C,** GSEA based on KEGG pathways in CSC-signature^high^ GBM cells. **D,** mRNA expression of MAPK family members in CSC-signature^high^ GBM cells. **E,** GSEA of ERK5 pathway in CSC-signature^high^ GBM cells. **F,** TGS-01 and TGS-04 cells were cultured in neurosphere medium or adherent culture medium, followed by determination of protein levels of ERK5, SOX2, and c-MYC (β-ACTIN served as a loading control; **G**), protein levels of nuclear ERK5 (LaminB1 served as the loading control; **H**), and mRNA levels of *SOX2*, *c-MYC*, and *ERK5* in TGS-01 and TGS-04 cells (**I**; *n* = 4, mean ± SE; *, *P* < 0.05; **, *P* < 0.01).

We first analyzed a scRNA-seq dataset of clinical GBM specimens to determine the properties of GSCs (24). We defined *EGFR*^+^ cells as GBM cells as per a previous study (24). We then divided the GBM cell population into two clusters: CSC-signature^high^ GBM cells and CSC-signature^low^ GBM cells, based on a ssGSEA (Fig. 1A). We confirmed that gene sets involved in stemness and stem cell were significantly enriched in CSC-signature^high^ GBM cells, allowing us to define these cells as the GSC population (Fig. 1B). CSC-signature^high^ GBM cells significantly upregulated a gene panel involved in the MAPK signaling pathway (Fig. 1C). Moreover, gene expression profiling of the MAPK family showed that the expression level of *ERK5* was significantly upregulated in CSC-signature^high^ GBM cells compared with CSC-signature^low^ GBM cells. In contrast, expression levels of other MAPK members in the CSC-signature^high^ GBM cells were indistinguishable from those in CSC-signature^low^ GBM cells (Fig. 1D). Similarly, gene set involved in the ERK5 pathway was significantly enriched in CSC-signature^high^ GBM cells, suggesting that ERK5 is related to stem cell properties in GSCs (Fig. 1E). We also analyzed different scRNA-seq datasets of clinical GBM specimens. We confirmed that the GSC pool was characterized by the elevated expression of *H2AFZ* and significant upregulation of the stemness-related genes, and differentiated GBM cells showed high expression of canonical differentiation marker, *GFAP* in accordance with a previous report (ref. 25; Supplementary Fig. S1A–S1C). In this dataset, we also observed that GSCs significantly upregulated gene panels related to MAPK and ERK5 pathways (Supplementary Fig. S1D and S1E).

Furthermore, we compared the ERK5 expression level between GSCs and differentiated glioma cells. TGS-01 and TGS-04 cells, which are human GBM patient-derived GSCs, were cultured in neurosphere culture condition (for GSCs) or adherent culture condition (for differentiated glioma cells; Fig. 1F). Consistent with results from our bioinformatics analysis, protein levels of ERK5 were increased in TGS-01 and TGS-04 GSCs, concomitant with higher levels of stem cell transcription factors, SOX2 and c-MYC (Fig. 1G). In addition, the nuclear abundance of ERK5 was also increased in TGS-01 and TGS-04 GSCs (Fig. 1H). Moreover, mRNA levels of *SOX2* and *c-MYC* were significantly increased in TGS-01 and TGS-04 GSCs, along with a significant reduction of *ERK5* (Fig. 1I). Thus, high expression levels of ERK5 were preferentially present in GSCs, suggesting that ERK5 might have a potential role in regulating the stem cell phenotypes of GSCs.

### Disrupting *ERK5* Impairs the Self-renewal Potential of GSCs *In Vitro*

**FIGURE 2:**
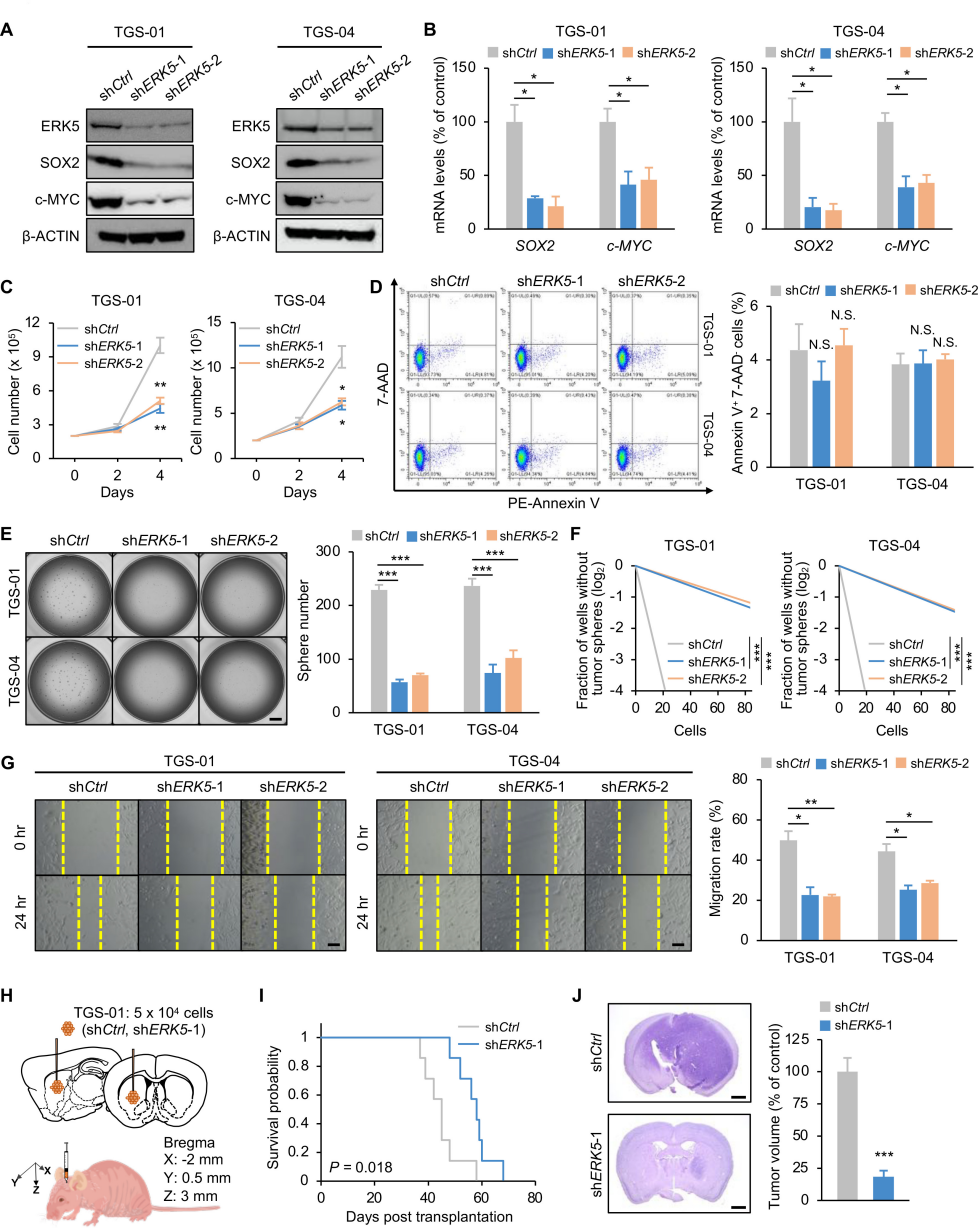
Disrupting *ERK5* impairs the self-renewal potential and the tumorigenicity of GSCs. TGS-01 and TGS-04 cells were infected with sh*ERK5*, followed by determination of protein levels of ERK5, SOX2, and c-MYC (β-ACTIN served as the loading control; **A**), mRNA levels of *SOX2* and *c-MYC* (*n* = 4, mean ± SE; *, *P* < 0.05; **B**), cell viability (*n* = 4, mean ± SE; *, *P* < 0.05; **, *P* < 0.01; **C**), cell apoptosis (*n* = 3, mean ± SE; **D**), tumorsphere number (*n* = 5, mean ± SE; ***P < 0.001; **E**), stem cell frequency by *in vitro* limiting dilution assay (estimated frequencies of clonogenic cells in GSC tumorspheres were calculated by extreme limiting dilution analysis (ELDA); ***, *P* < 0.001; **F**), and migration ability (*n* = 4, mean ± SE; *, *P* < 0.05; **, *P* < 0.01; **G**). **H** and **I,** Kaplan–Meier survival analysis of mice inoculated with TGS-01 cells infected with sh*ERK5* (*n* = 7). *P* value was calculated using a log-rank test. **I,** Histologic analyses of brains dissected 40 days after intracranial transplantation. Tissue sections were stained with H&E (*n* = 5, mean ± SE; ***, *P* < 0.001). Scale bars, 1 mm (**E** and **J**) and 100 μm (**G**).

We next proved the functional importance of ERK5 in the maintenance of GSCs *in vitro* by targeting *ERK5* expression using lentiviral short hairpin RNA (shRNA; sh*ERK5*) in TGS-01 and TGS-04 GSCs. *ERK5* knockdown exhibited a marked reduction in protein levels of SOX2 and c-MYC in both TGS-01 and TGS-04 GSCs, along with a significant reduction in their mRNA levels (Fig. 2A and B). *ERK5* silencing remarkably reduced cell proliferation of both TGS-01 and TGS-04 GSCs (Fig. 2C). Annexin V staining showed that targeting *ERK5* did not significantly alter cell apoptosis in both TGS-01 and TGS-04 GSCs (Fig. 2D). A tumorsphere formation assay indicated that silencing *ERK5* significantly reduced GSC tumorsphere formation in both TGS-01 and TGS-04 GSCs (Fig. 2E). Furthermore, an *in vitro* dilution assay demonstrated that the self-renewal potential of GSCs was significantly impaired by *ERK5* silencing in both TGS-01 and TGS-04 GSCs (Fig. 2F). In addition, *ERK5* disruption decreased the migration potential significantly, as demonstrated by the wound healing assay in both TGS-01 and TGS-04 GSCs (Fig. 2G). These data suggest that ERK5 is required for the self-renewal potential and aggressiveness of GSCs *in vitro*.

### Targeting *ERK5* Abrogates the Tumorigenicity of GSCs *In Vivo*

Considering that ERK5 is required for cell growth, aggressiveness, and self-renewal of GSCs, we next elucidated whether targeting ERK5 could affect the tumorigenic potential of GSCs in an orthotopic xenograft mouse model (Fig. 2H). Equal numbers of TGS-01 GSCs infected with sh*ERK5* or sh*Control* (sh*Ctrl*) were intracranially injected into immunocompromised mice. The mice inoculated with sh*ERK5* infected TGS-01 GSCs had significantly longer survival than those inoculated with sh*Ctrl* infected TGS-01 GSCs (Fig. 2I). In addition, histologic examination demonstrated that the mice inoculated with sh*ERK5*-infected TGS-01 GSCs displayed a significant reduction in intracranial tumor growth compared with sh*Ctrl*-infected TGS-01 GSCs (Fig. 2J). Attenuated tumor growth of GSCs was also observed by *ERK5* knockdown using shRNA targeting different regions of the gene sequence (Supplementary Fig. S2A and S2B). These results demonstrate that ERK5 contributes to maintenance of the tumorigenic capacity of GSCs *in vivo*.

### The MEK5-ERK5 Axis Promotes the Self-renewal Potential of GSCs *In Vitro*

**FIGURE 3:**
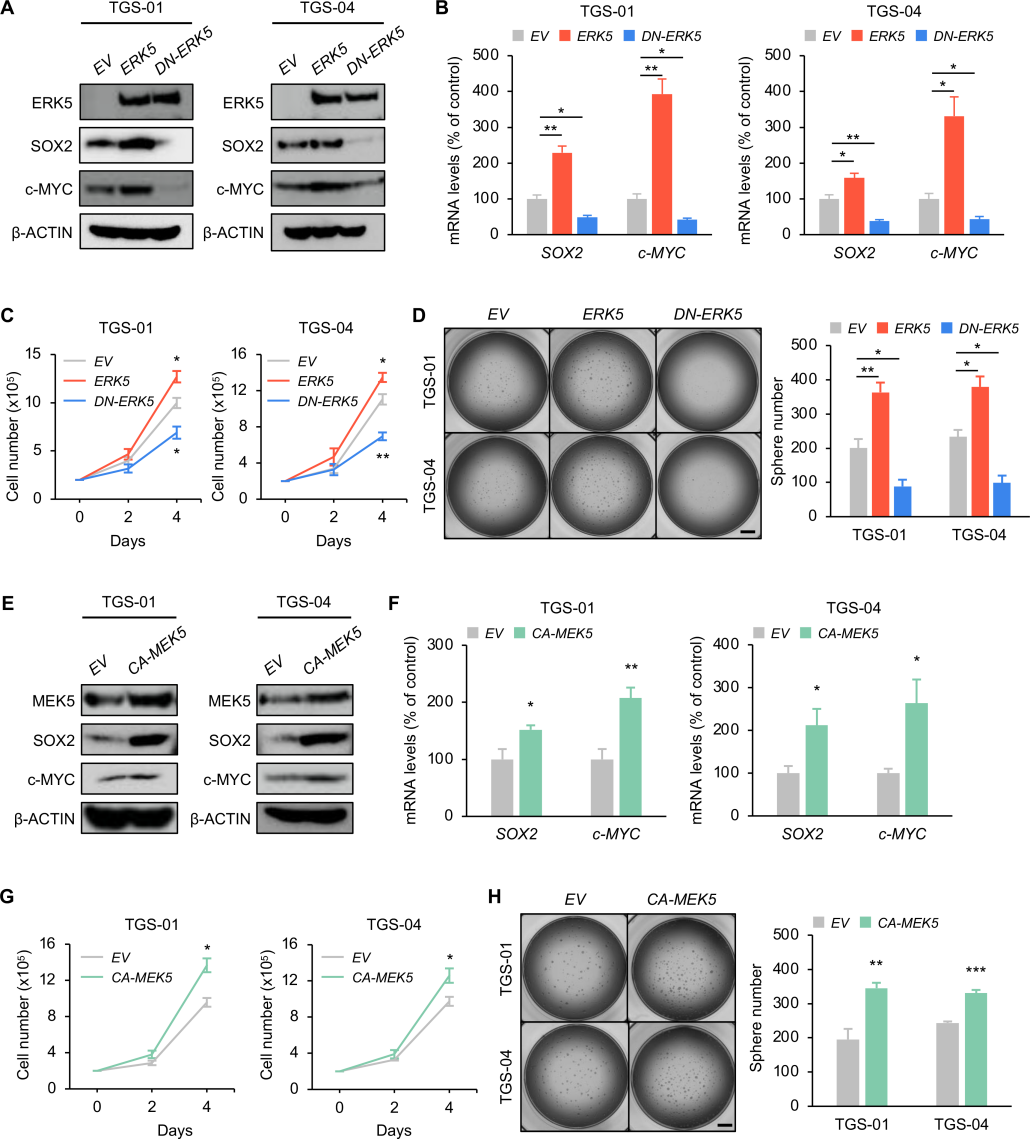
The MEK5-ERK5 pathway potentiates the self-renewal activity of GSCs *in vitro.* TGS-01 and TGS-04 cells were infected with *ERK5* and *DN-ERK5* expression vectors, followed by determination of protein levels of ERK5, SOX2, and c-MYC (β-ACTIN served as the loading control; **A**), mRNA levels of *SOX2* and *c-MYC* (*n* = 4, mean ± SE; *, *P* < 0.05; **, *P* < 0.01; **B**), cell viability (*n* = 4, mean ± SE; *, *P* < 0.05; **, *P* < 0.01; **C**), and tumorsphere number (*n* = 4–5, mean ± SE; *, *P* < 0.05; **, *P* < 0.01; **D**). TGS-01 and TGS-04 cells were infected with *CA-MEK5* expression vector, followed by determination of protein levels of MEK5, SOX2, and c-MYC (β-ACTIN served as the loading control; **E**), mRNA levels of *SOX2* and *c-MYC* (*n* = 4, mean ± SE; *, *P* < 0.05; **, *P* < 0.01; **F**), cell viability (*n* = 4, mean ± SE; *, *P* < 0.05; **G**), and tumorsphere number (*n* = 5, mean ± SE; **, *P* < 0.01; ***, *P* < 0.001; **H**). Scale bar, 1 mm (**D** and **H**).

We next investigated the effect of the MEK5-ERK5 axis on the cell growth and self-renewal capacity of GSCs. *ERK5* overexpression markedly upregulated protein levels of SOX2, c-MYC, and nuclear ERK5 in both TGS-01 and TGS-04 GSCs, along with a significant increase in their mRNA levels, while *DN-ERK5* (the dominant negative form of ERK5 that lacks two phosphorylation sites for MEK5 and cannot phosphorylate target genes) overexpression markedly decreased SOX2, c-MYC, and nuclear ERK5 protein levels, concomitant with a significant reduction in their mRNA levels (Fig. 3A and B; Supplementary Fig. S3A). Contrary to the suppressive effect of *ERK5* knockdown on GSC properties (Fig. 2C and E), *ERK5* overexpression significantly increased cell proliferation and tumorsphere formation in both TGS-01 and TGS-04 GSCs (Fig. 3C and D). In contrast, the introduction of *DN-ERK5* decreased cell proliferation and tumorsphere formation in both TGS-01 and TGS-04 GSCs (Fig. 3C and D), consistent with our prior studies on *ERK5* silencing GSCs (Fig. 2C and E).

To further confirm the functional significance of the MEK5-ERK5 axis in the regulation of GSC stemness, *constitutively active-MEK5* (*CA-MEK5*) was overexpressed in both TGS-01 and TGS-04 GSCs (Fig. 3E). We confirmed that *CA-MEK5* increased nuclear abundance of ERK5 in both TGS-01 and TGS-04 GSCs (Supplementary Fig. S3B). *CA-MEK5* overexpression significantly increased cell proliferation and tumorsphere formation concomitant with an increased expression of SOX2 and c-MYC at protein and mRNA levels (Fig. 3E–H). Thus, the MEK5-ERK5 axis could regulate the self-renewal potential of GSCs *in vitro*.

### ERK5 Regulates the Self-renewal Potential of GSCs Partly Through STAT3

**FIGURE 4:**
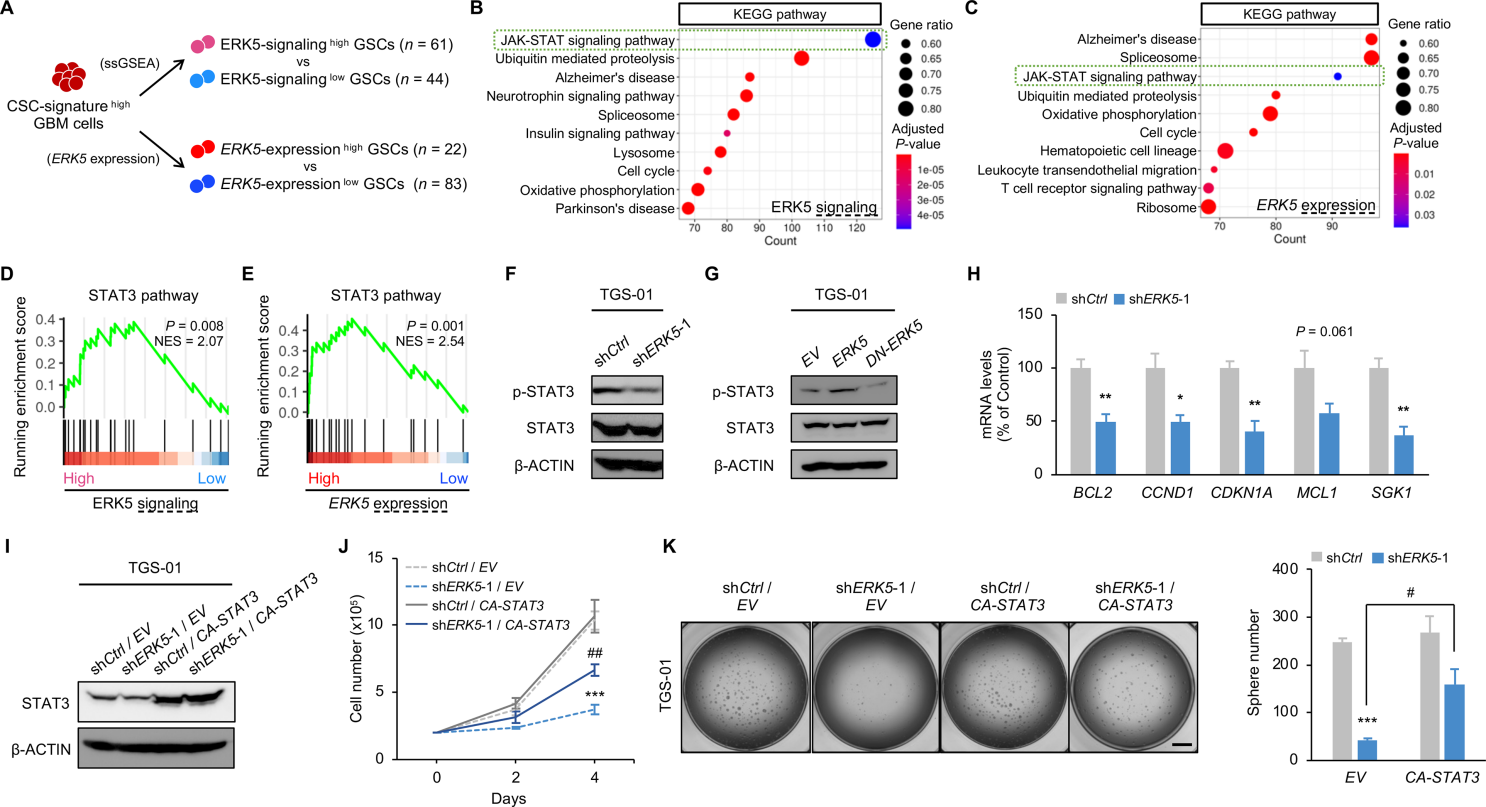
Overexpression of *STAT3* restores the suppressive effect of *ERK5* silencing on GSC phenotypes. **A,** Experimental schemes for scRNA-seq of CSC-signature^high^ GBM cells. GSEA based on KEGG pathways in ERK5-signaling^high^ GSCs (**B**) and *ERK5*-expression^high^ GSCs (**C**). GSEA of STAT3 pathway in ERK5-signaling^high^ GSCs (**D**) and *ERK5*-expression^high^ GSCs (**E**). Protein levels of p-STAT3 and STAT3 in TGS-01 cells infected with sh*ERK5* (**F**), *ERK5,* and *DN-ERK5* expression vectors (β-ACTIN served as the loading control; **G**). **H,** mRNA levels of STAT3 target genes in TGS-01 cells infected with sh*ERK5* vector (*n* = 4, mean ± SE; *, *P* < 0.05; **, *P* < 0.01). TGS-01 cells were infected with sh*ERK5* in combination with *CA-STAT3* expression vector, followed by determination of protein levels of STAT3 (β-ACTIN served as a loading control; **I**), cell viability (*n* = 4, mean ± SE; ***, *P* < 0.001; ^##^, *P* < 0.01; **J**), and tumorsphere number (*n* = 5, mean ± SE; ***, *P* < 0.001; ^#^, *P* < 0.05; **K**). Scale bar, 1 mm (**K**).

We next examined the molecular mechanisms of how ERK5 controls the maintenance of GSCs. We subclustered CSC-signature^high^ GBM cells to identify ERK5-signaling^high^ GSCs and *ERK5*-expression^high^ GSCs, based on a ssGSEA and the expression level of *ERK5*, respectively (Fig. 4A). Kyoto Encyclopedia of Genes and Genomes (KEGG) pathway mapping revealed that a gene set involved in the JAK-STAT signaling pathway was significantly enriched in both ERK5-signaling^high^ GSCs and *ERK5*-expression^high^ GSCs (Fig. 4B and C).

STAT3, which is a transcriptional factor, is associated with poor prognosis in patients with GBM (27). Furthermore, previous studies reported that the STAT3 pathway is constitutively activated in GSCs, and the activation of STAT3 is critical for maintaining the stemness of GSCs (28). Moreover, a recent study showed that *ERK5* deficiency reduced the level of STAT3 phosphorylation at Tyr705 (p-STAT3^Y705^), which is the active form of STAT3, in macrophages (29). These reports suggest the possibility that STAT3 is a downstream factor of ERK5 regulating GSC stemness. Indeed, STAT3 pathway-related genes were significantly upregulated in both ERK5-signaling^high^ GSCs and *ERK5*-expression^high^ GSCs (Fig. 4D and E). Likewise, the analysis of different scRNA-seq dataset recapitulated the upregulation of STAT3 pathway-related genes in both ERK5-signaling^high^ GSCs and *ERK5*-expression^high^ GSCs (Supplementary Fig. S4A–S4C). Moreover, consistent with our bioinformatics analysis, we confirmed the reduction of STAT3^Y705^ phosphorylation levels in *ERK5-*silenced TGS-01 GSCs and *DN-ERK5–*overexpressing TGS-01 GSCs (Fig. 4F). Meanwhile, *ERK5* and *CA-MEK5* overexpression increased STAT3^Y705^ phosphorylation levels in TGS-01 GSCs (Fig. 4G; Supplementary Fig. S4D). STAT3^Y705^ phosphorylation levels were also increased in TGS-01 GSCs compared with differentiated TGS-01 GBM cells (Supplementary Fig. S4E). Thus, STAT3 target genes were downregulated in *ERK5* silenced TGS-01 GSCs (Fig. 4H). Furthermore, the introduction of *CA-STAT3* restored cell proliferation and rescued the impaired tumorsphere formation induced by silencing of *ERK5* in TGS-01 GSCs (Fig. 4I–K). These results indicate that ERK5 mediates the STAT3 pathway to regulate GSC self-renewal potential.

### ERK5 is Associated with Poor Prognosis of Patients with GBM with High Stem Cell Properties

**FIGURE 5:**
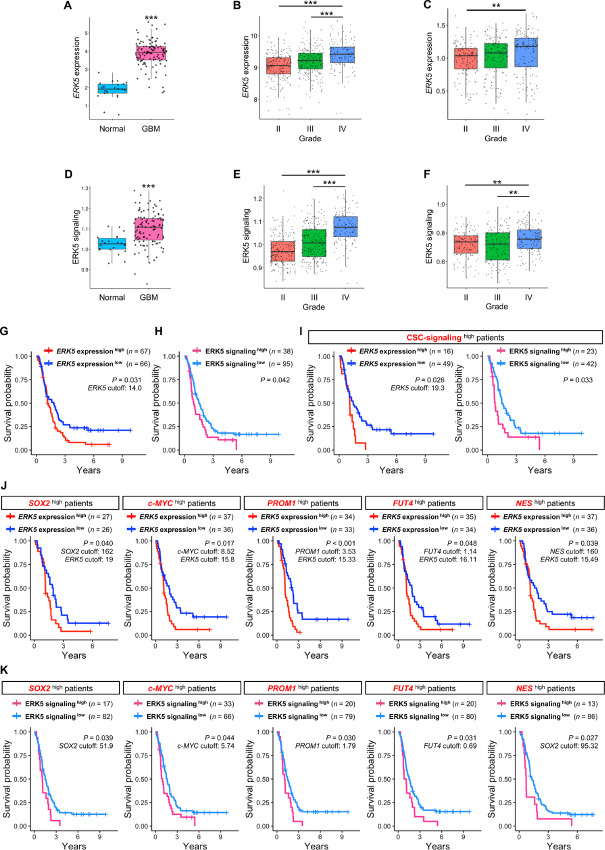
ERK5 has an impact on survival of patients with GBM with high stem cell properties. **A,** mRNA expression of *ERK5* in GBM tissues (*n* = 107) and normal tissues (*n* = 22) in a previous study (26). **B,** mRNA expression of *ERK5* at each grade (grade II, *n* =  226; grade III, *n* =  241; grade IV, *n* =  149) in TCGA database. **C,** mRNA expression of *ERK5* at each grade (grade II, *n* =  138; grade III, *n* =  144; grade IV, *n* =  140) in CGGA database. **D,** ssGSEA score of ERK5 signaling in GBM tissues (*n* = 107) and normal tissues (*n* = 22) in our previous data. **E,** ssGSEA score of ERK5 signaling at each grade (grade II, *n* =  226; grade III, *n* =  241; grade IV, *n* =  149) in TCGA database. **F,** ssGSEA score of ERK5 signaling at each grade (grade II, *n* =  138; grade III, *n* =  144; grade IV, *n* =  140) in CGGA database. Kaplan–Meier survival analysis of *ERK5* expression^high^ (*n* = 67) and *ERK5* expression^low^ (*n* = 66; **G**), ERK5 signaling^high^ (*n* = 38) and ERK5 signaling^low^ (*n* = 95; **H**), GSC signaling^high^ (*ERK5* expression^high^ (*n* = 17), *ERK5* expression^low^ (*n* = 53); ERK5 signaling^high^ (*n* = 23), ERK5 signaling^low^ (*n* = 47; **I**), *SOX2*^high^ [*ERK5* expression^high^ (*n* = 27) and *ERK5* expression^low^ (*n* = 26)], *c-MYC*^high^ [*ERK5* expression^high^ (*n* = 37) and *ERK5* expression^low^ (*n* = 36)], *PROM1*^high^ [*ERK5* expression^high^ (*n* = 34) and *ERK5* expression^low^ (*n* = 33)], *FUT4*^high^ [*ERK5* expression^high^ (*n* = 35) and *ERK5* expression^low^ (*n* = 34)], *NES*^high^ [*ERK5* expression^high^ (*n* = 37) and *ERK5* expression^low^ (*n* = 36; **J**), and *SOX2*^high^ [ERK5 signaling^high^ (*n* = 17) and ERK5 signaling^low^ (*n* = 82)], *c-MYC*^high^ [ERK5 signaling^high^ (*n* = 33) and ERK5 signaling^low^ (*n* = 66)], *PROM1*^high^ [ERK5 signaling^high^ (*n* = 20) and ERK5 signaling^low^ (*n* = 79)], *FUT4*^high^ [ERK5 signaling^high^ (*n* = 20) and ERK5 signaling^low^ (*n* = 80)], *NES*^high^ [ERK5 signaling^high^ (*n* = 13) and ERK5 signaling^low^ (*n* = 86)] GBM patient groups in CGGA database (**K**).

To determine the clinical relevance of our findings, we performed *in silico* studies on a publicly available clinical datasets in patients with GBM (26). *ERK5* expression and ERK5 signaling were significantly upregulated in GBM tissues compared with non-tumor brain tissues and were associated with increased glioma grade (Fig. 5A–F). *ERK5* expression does not differ regardless of MGMT methylation status, *IDH* mutation status, age, and sex (Supplementary Fig. S5A–S5D and S5F–S5I). *ERK5* expression was significantly downregulated in the neural subtype in TCGA database while no marked alterations of *ERK5* expression were found among classical, mesenchymal, and proneural subtypes in the CGGA database (Supplementary Fig. S5E and S5J). The Kaplan–Meier survival analysis demonstrated that patients with GBM with higher *ERK5* expression had significantly shorter survival durations than those with low expression (Fig. 5G), which is consistent with a previous study. Likewise, patients with GBM with elevated ERK5 signaling also displayed a significantly short survival duration (Fig. 5H).

Previous studies indicated that ERK5 expressed by GSCs plays a critical role in maintaining stemness and tumorigenicity in GSCs; thus, we assessed whether *ERK5* in patients with GBM harboring higher stem cell properties is associated with poor prognosis. We found that high expression of *ERK5* and elevated ERK5 signaling were associated with poor prognosis in GSC signaling^high^ patients (Fig. 5I). Furthermore, high expression of *ERK5* and increased ERK5 signaling are associated with poor prognosis in patients harboring high expression of stemness markers such as *SOX2*, *c-MYC*, *PROM1*, *FUT4*, and *NES* (Fig. 5J and K). In contrast, we found that *ERK5* expression and ERK5 signaling do not correlate with poor prognosis in patients with lower stem cell properties (Supplementary Fig. S6A–S6C). The human GBM clinical data suggest that ERK5 expressed in GSCs, rather than differentiated GBM cells, could affect the survival of patients with GBM.

### The Pharmacologic Inhibition of ERK5 Suppresses the Self-renewal Potential and Tumorigenicity of GSCs

**FIGURE 6:**
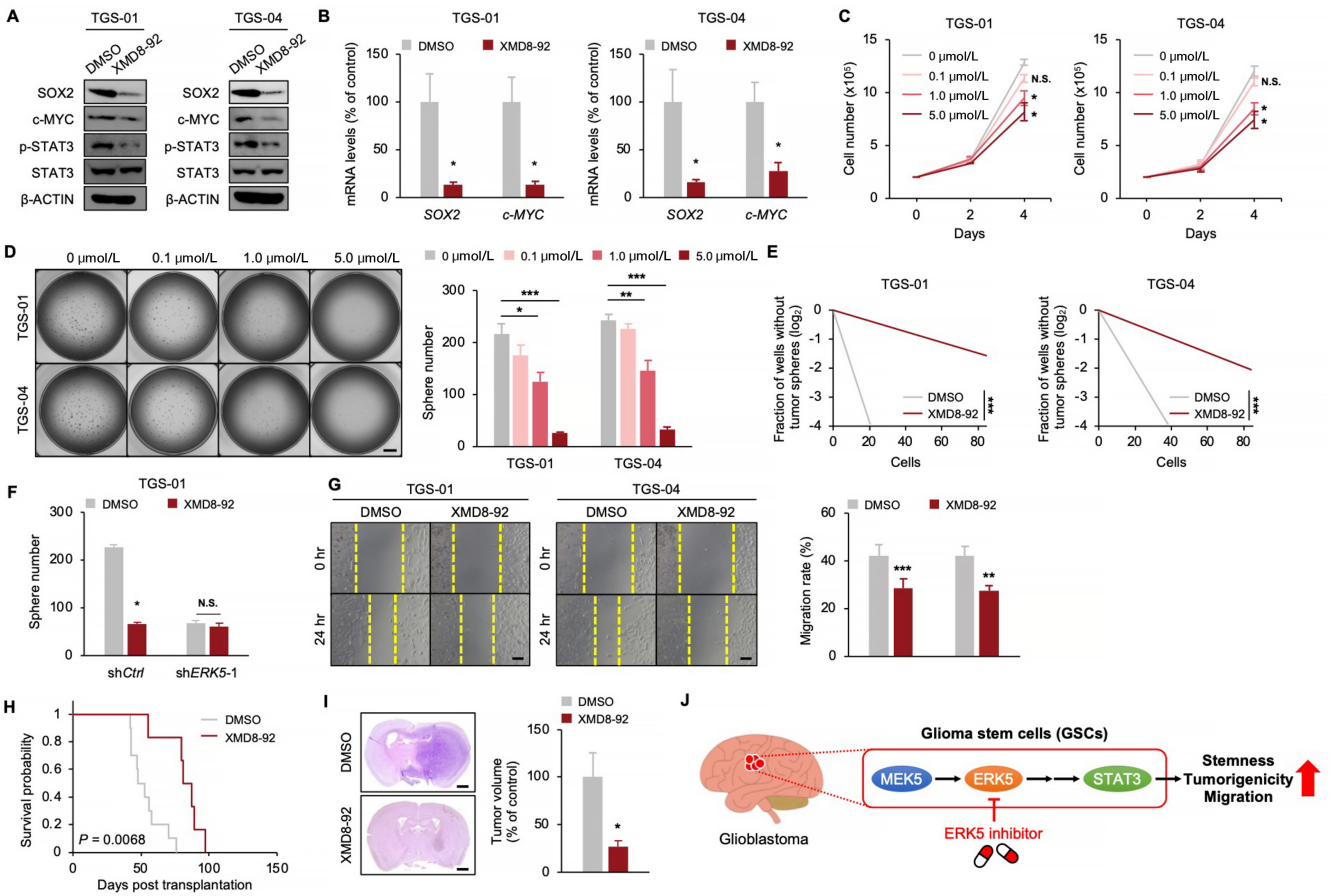
The pharmacologic inhibition of ERK5 suppresses GSC phenotypes. TGS-01 and TGS-04 cells were treated with XMD8-92, followed by determination of protein levels of SOX2, c-MYC, p-STAT3, and STAT3 (β-ACTIN served as the loading control; **A**), mRNA levels of *SOX2* and *c-MYC* (*n* = 4, mean ± SE; *, *P* < 0.05; **B**), cell viability (*n* = 4, mean ± SE; *, *P* < 0.05; **C**), tumorsphere number (*n* = 10, mean ± SE; *, *P* < 0.05; **, *P* < 0.01; ***, *P* < 0.001; **D**), stem cell frequency by *in vitro* limiting dilution assay (estimated frequencies of clonogenic cells in GSC tumorspheres were calculated by ELDA; ***, *P* < 0.001; **E**). **F,** TGS-01 cells were infected with sh*ERK5* in combination with XMD8-92 treatment at 5.0 μmol/L followed by determination of tumorsphere number (*n* = 5, mean ± SE; *, *P* < 0.05). Migration ability in TGS-01 cells infected with sh*ERK5* (*n* = 4, mean ± SE; **, *P* < 0.01; ***, *P* < 0.001; **G**), Kaplan–Meier survival analysis of mice inoculated with TGS-01 cells pre-treated with 5.0 μmol/L KY-065 for 4 days (*n* = 6–10; **H**). *P* value was calculated using a log-rank test. **I,** Histologic analyses of brains dissected at 40 days after intracranial transplantation. Tissue sections were stained with H&E (*n* = 5, mean ± SE; *, *P* < 0.05). **J,** Schematic model of the findings of this study. MEK5-ERK5-STAT3 axis in GSCs promotes stemness, tumorigenicity, and migration to enhance GBM malignancy. Pharmacologic inhibition of ERK5 by XMD8-92 inhibits this pathway, providing a potential strategy for GSC-directed therapy. Scale bars, 1 mm (**D** and **I**) and 100 μm (**G**).

Our studies on patient-derived GSCs *in vitro* and *in vivo* and GBM clinical specimens indicated that ERK5 expressed in GSCs could be a promising target for GBM therapy. For clinical application of our findings, we determined whether pharmacologic inhibition of ERK5 by XMD8-92, a small-molecule inhibitor of ERK5 (20, 21, 30), could suppress the self-renewal potential and tumorigenicity of GSCs; 5.0 μmol/L of XMD8-92 treatment decreased protein levels of SOX2 and c-MYC, concomitant with a reduction in the phosphorylation level of STAT3^Y705^ in both TGS-01 and TGS-04 GSCs (Fig. 6A). Moreover, mRNA levels of *SOX2* and *c-MYC* were significantly downregulated by XMD8-92 treatment at 5.0 μmol/L in both TGS-01 and TGS-04 GSCs (Fig. 6). XMD8-92 significantly decreased cell proliferation and tumorsphere number in a dose-dependent manner over time in both TGS-01 and TGS-04 GSCs (Fig. 6C and D). Furthermore, XMD8-92 treatment at 5.0 μmol/L significantly decreased the GSC self-renewal activity (Fig. 6E). No additional decrease in the tumorsphere number was observed following XMD8-92 treatment at 5.0 μmol/L in sh*ERK5*-infected GSCs (Fig. 6F). XMD8-92 treatment at 5.0 μmol/L suppressed the GSC migratory capacity (Fig. 6G). In addition, treatment with a different ERK5 inhibitor, AX15836 (31) recapitulated the GSC phenotypes observed in XMD8-92 treatment-mediated ERK5 inhibition (Supplementary Fig. S7A–S7E).

We examined whether XMD8-92 could disrupt tumor growth in GSC-derived orthotopic xenografts. The survival of mice inoculated with TGS-01 GSCs, transiently pretreated with 5.0 μmol/L of XMD8-92 was significantly prolonged, with significant inhibition of GSC-driven tumor growth (Fig. 6H and I). These data demonstrate that XMD8-92 treatment effectively inhibits the self-renewal potential and tumorigenicity of GSCs, thus, exerting anti-GBM potential.

## Discussion

Previous studies reported that STAT3 functions to regulate gene networks involved with maintaining stem cell phenotypes in CSCs (32). Moreover, STAT3 upregulates CSC markers such as CD44, leading to the promotion of CSC properties (33). In addition, STAT3 plays an important role in controlling self-renewal, survival, and stem cell transformation in GSCs (34, 35). Aberrant STAT3 activation is preferentially present in GSCs relative to differentiated GBM cells, and its inhibition suppresses the self-renewal capacity of GSCs (36), suggesting that inhibition of the STAT3 signaling pathway may have significant therapeutic potential. However, the specific inhibition of STAT3 has been challenging in clinical use because STAT3 is required for fundamental cellular functions in normal cells (37). Thus, a comprehensive understanding of the molecular mechanism of STAT3 activation may provide new GSC-specific therapeutic targets to improve GBM treatment. In this study, we demonstrated that ERK5 mediates phosphorylation of STAT3^Y705^ to exert its function in GSCs. Our study adds ERK5 to the list of upstream factors such as BMX, CD9, IL6, Notch, PDGFR, and PI3K, to activate STAT3 pathway in GSCs (34, 38–42), suggesting that targeting ERK5 could be an alternative way to suppress the STAT3 pathway to inhibit the self-renewal potential in GSCs.

We found that genetic or pharmacologic inhibition of ERK5 decreases the level of STAT3^Y705^ phosphorylation in GSCs to control stem cell phenotypes. However, how ERK5 modulates the phosphorylation of STAT3^Y705^ in GSCs remains unclear and more mechanistic insights are needed. Because ERK5 is a serine/threonine kinase, ERK5 should indirectly increase the level of STAT3^Y705^ phosphorylation through other signaling pathways. A recent study demonstrated that SOCS3, a classic negative regulator of the JAK2-STAT3 pathway, was upregulated by the inactivation of ERK5 (29). Moreover, ERK5 is required for sustained AKT pathway activation upon stimulation of PDGFR (43). These pathways regulate STAT3^Y705^ phosphorylation in GSCs to control stem cell renewal potential (44, 45). Thus, these pathways may be involved in the mechanism by which ERK5 modulates the phosphorylation of STAT3^Y705^ to maintain stemness in GSCs. Meanwhile, the ectopic expression of *STAT3* did not perfectly rescue the impairment of stem cell renewal activity in *ERK5*-silenced GSCs. We could not exclude the possibility that additional pathways may also partly mediate the function of ERK5 in GSCs. Therefore, further studies are required to clarify the mechanisms by which ERK5 regulates the tumorigenesis of GSCs.

Beyond the role in inhibiting tumor initiation and progression, targeting ERK5 has the potential to augment the effectiveness of chemotherapy and radiotherapy. For example, a recent study demonstrated that temozolomide treatment combined with ERK5 inhibition leads to a significant increase in DNA damage *in vitro* experiments using non-stem GBM cells (22). Pharmacologic inhibition of ERK5 by XMD8-92 promotes the effect of 5-fluorouracil–based chemotherapy in colon cancer cells (17). Moreover, ERK5 inhibition sensitized cancer cells to radiotherapy by suppressing the ability to repair radiation-induced DNA damage in lung and prostate cancer cells (46). Given these reports and our findings presented here, it would be interesting to test whether ERK5 inhibition could synergize with chemotherapy and radiotherapy to disrupt GSC maintenance and overcome the therapeutic resistance of GSCs.

In conclusion, our studies identified the MEK5-ERK5-STAT3 axis as a critical regulator maintaining the self-renewal and tumorigenic potential of GSCs. Moreover, we confirmed that pharmacological inhibition of ERK5 by XMD8-92 attenuated the self-renewal ability, tumorigenicity, and migratory capacity of GSCs (Fig. 6J). To our knowledge, this is the first preclinical study to examine the functional role of ERK5 in CSC tumorigenesis *in vivo*. Our findings provide new insights into the molecular mechanism driving CSC proliferation and self-renewal. Our data also suggest that targeting the MEK5-ERK5-STAT3 axis could be an effective therapeutic approach against various cancers whose malignancies are connected to the stemness of CSCs.

## Supplementary Material

Supplementary Table ST1 Supplementary Table

Figure S1 Figure S1

Figure S2 Figure S2

Figure S3 Figure S3

Figure S4 Figure S4

Figure S5 Figure S5

Figure S6 Figure S6

Figure S7 Figure S7
