# Supplementary material for: MEK5-ERK5 Axis Promotes Self-renewal and Tumorigenicity of Glioma Stem Cells
Source: Cancer Res Commun. 2023 Jan 30;3(1):148–59. doi: 10.1158/2767-9764.CRC-22-0243 (PMC10035453; doi:10.1158/2767-9764.CRC-22-0243)
Supplement: Supplementary Table ST1 — Supplementary Table [file crc-22-0243-s01.docx]

**Supplementary Table**

**List of primers used for real-time PCR.**

| **Gene** | **Forward (5’-3’)** | **Reverse (5’-3’)** |
| --- | --- | --- |
| ***BCL2*** | ATCGCCCTGTGGATGACTGAGT | GCCAGGAGAAATCAAACAGAGGC |
| ***CCND1*** | TCTACACCGACAACTCCATC | TCTGGCATTTTGGAGAGGAA |
| ***CDKN1A*** | AGGTGGACCTGGAGACTCTCAG | TCCTCTTGGAGAAGATCAGCCG |
| ***c-MYC*** | CCTGGTGCTCCATGAGGAGAC | CACTGTCCAACTTGACCCTCTTG |
| ***ERK5*** | GAACGCTGGACTCGAATGG | GAGGACTGGTAGGTTGGACTGG |
| ***GAPDH*** | TGTTGCCATCAATGACCCCTT | CTCCACGACGTACTCAGCG |
| ***MCL1*** | AGAAAGCTGCATCGAACCAT | CCAGCTCCTACTCCAGCAAC |
| ***SGK1*** | GCTGAAATAGCCAGTGCCTTGG | GTTCTCCTTGCAGAGTCCGAAG |
| ***SOX2*** | CCAATCCCATCCACACTCAC | ACCCTCCCCAGGTTTTCTCT |
