# Supplementary material for: MEK5-ERK5 Axis Promotes Self-renewal and Tumorigenicity of Glioma Stem Cells
Source: Cancer Res Commun. 2023 Jan 30;3(1):148–59. doi: 10.1158/2767-9764.CRC-22-0243 (PMC10035453; doi:10.1158/2767-9764.CRC-22-0243)
Supplement: Figure S1 [file crc-22-0243-s02.pptx]

## Slide 1
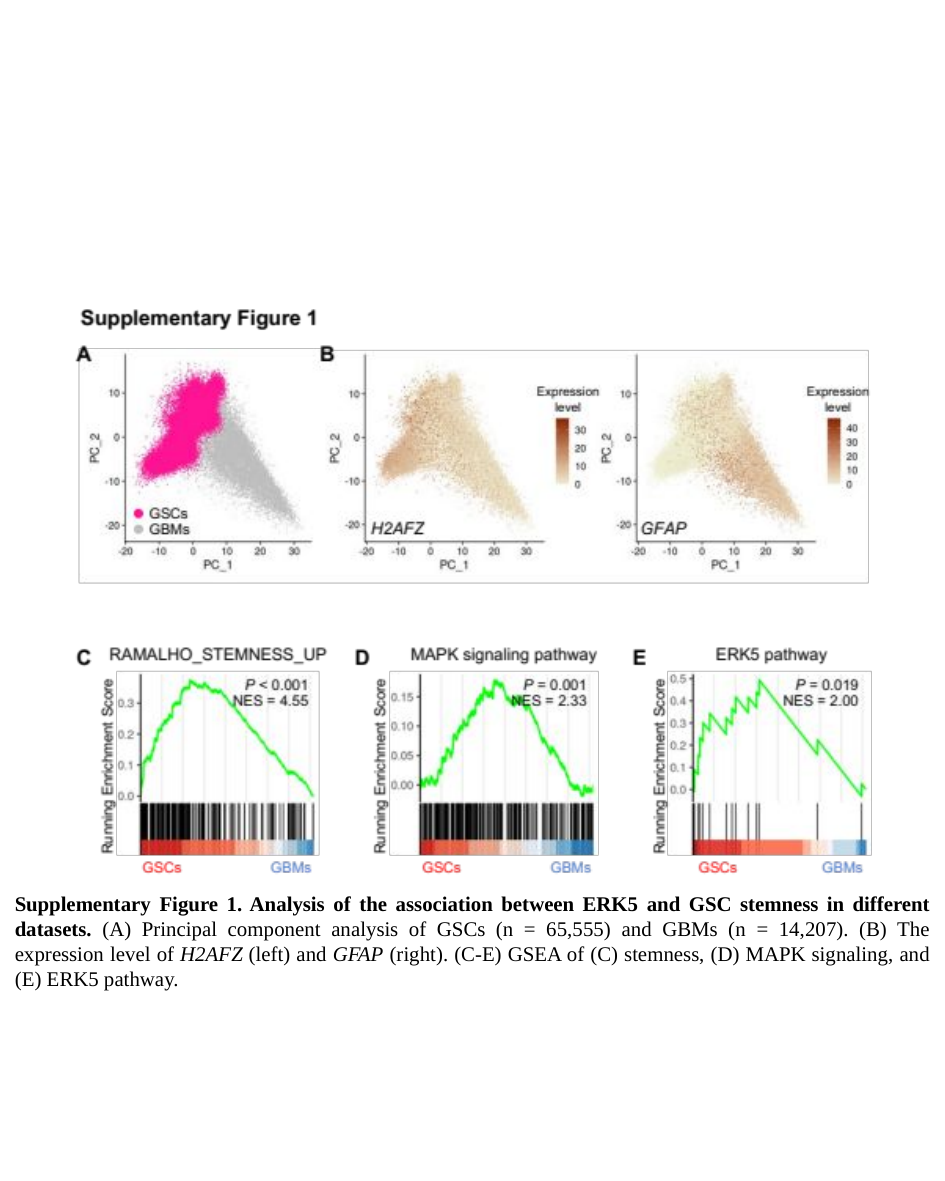

Supplementary Figure 1. Analysis of the association between ERK5 and GSC stemness in different datasets. (A) Principal component analysis of GSCs (n = 65,555) and GBMs (n = 14,207). (B) The expression level of H2AFZ (left) and GFAP (right). (C-E) GSEA of (C) stemness, (D) MAPK signaling, and (E) ERK5 pathway.
