# Supplementary material for: MEK5-ERK5 Axis Promotes Self-renewal and Tumorigenicity of Glioma Stem Cells
Source: Cancer Res Commun. 2023 Jan 30;3(1):148–59. doi: 10.1158/2767-9764.CRC-22-0243 (PMC10035453; doi:10.1158/2767-9764.CRC-22-0243)
Supplement: Figure S2 [file crc-22-0243-s03.pptx]

## Slide 1
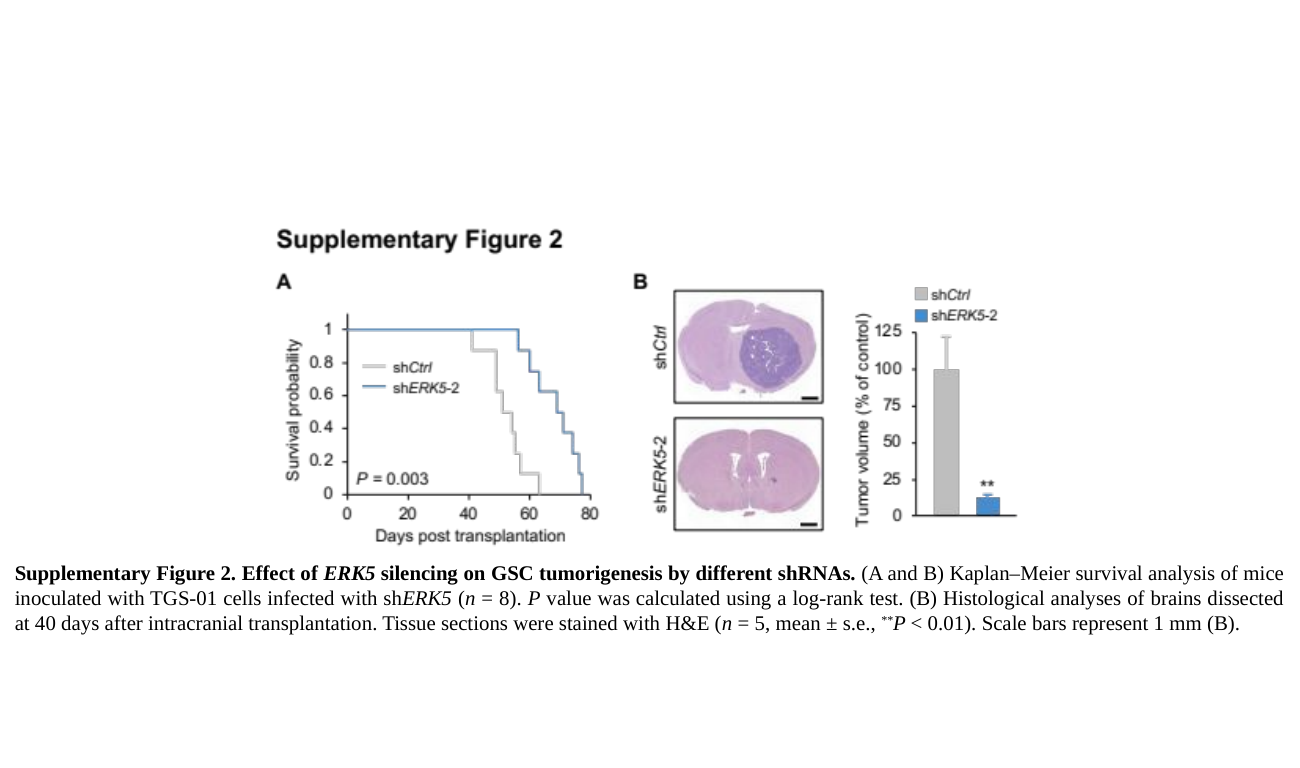

Supplementary Figure 2. Effect of ERK5 silencing on GSC tumorigenesis by different shRNAs. (A and B) Kaplan–Meier survival analysis of mice inoculated with TGS-01 cells infected with shERK5 (n = 8). P value was calculated using a log-rank test. (B) Histological analyses of brains dissected at 40 days after intracranial transplantation. Tissue sections were stained with H&E (n = 5, mean ± s.e., **P < 0.01). Scale bars represent 1 mm (B).
