# Supplementary material for: MEK5-ERK5 Axis Promotes Self-renewal and Tumorigenicity of Glioma Stem Cells
Source: Cancer Res Commun. 2023 Jan 30;3(1):148–59. doi: 10.1158/2767-9764.CRC-22-0243 (PMC10035453; doi:10.1158/2767-9764.CRC-22-0243)
Supplement: Figure S3 [file crc-22-0243-s04.pptx]

## Slide 1
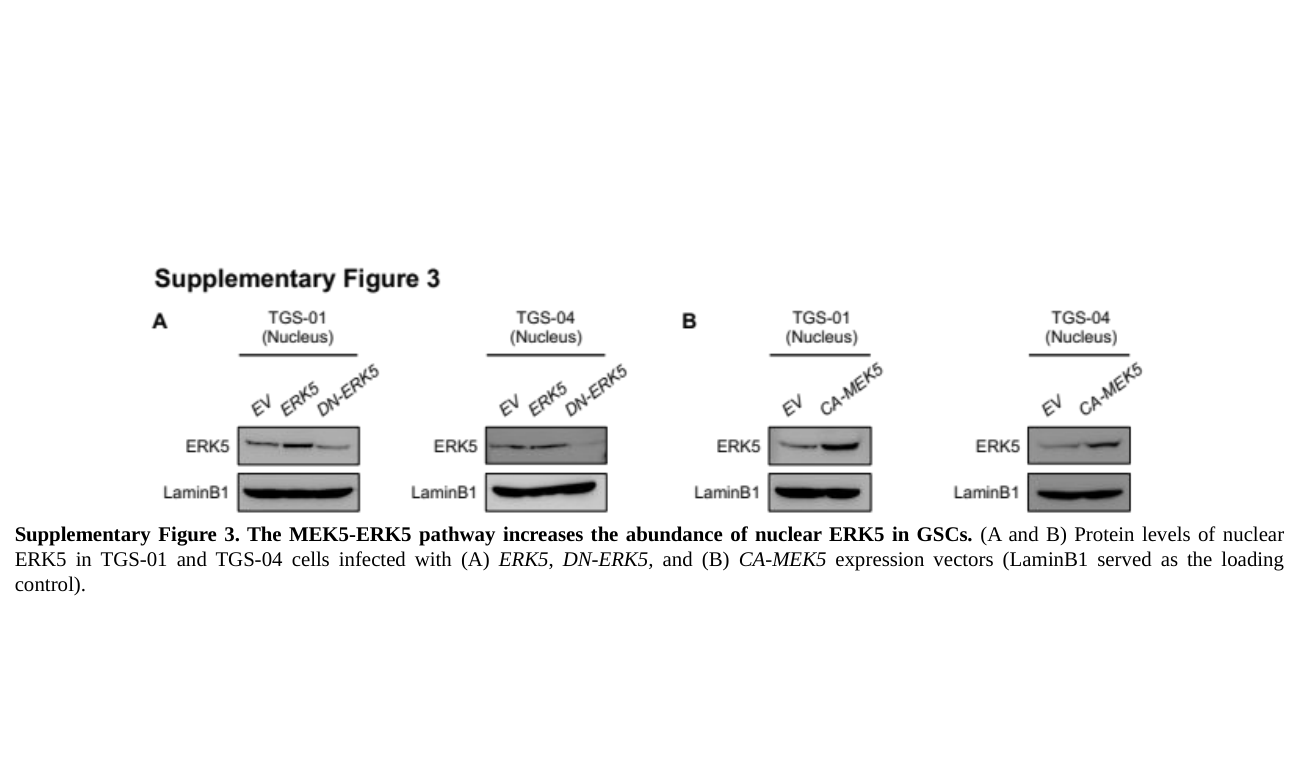

Supplementary Figure 3. The MEK5-ERK5 pathway increases the abundance of nuclear ERK5 in GSCs. (A and B) Protein levels of nuclear ERK5 in TGS-01 and TGS-04 cells infected with (A) ERK5, DN-ERK5, and (B) CA-MEK5 expression vectors (LaminB1 served as the loading control).
