# Supplementary material for: MEK5-ERK5 Axis Promotes Self-renewal and Tumorigenicity of Glioma Stem Cells
Source: Cancer Res Commun. 2023 Jan 30;3(1):148–59. doi: 10.1158/2767-9764.CRC-22-0243 (PMC10035453; doi:10.1158/2767-9764.CRC-22-0243)
Supplement: Figure S4 [file crc-22-0243-s05.pptx]

## Slide 1
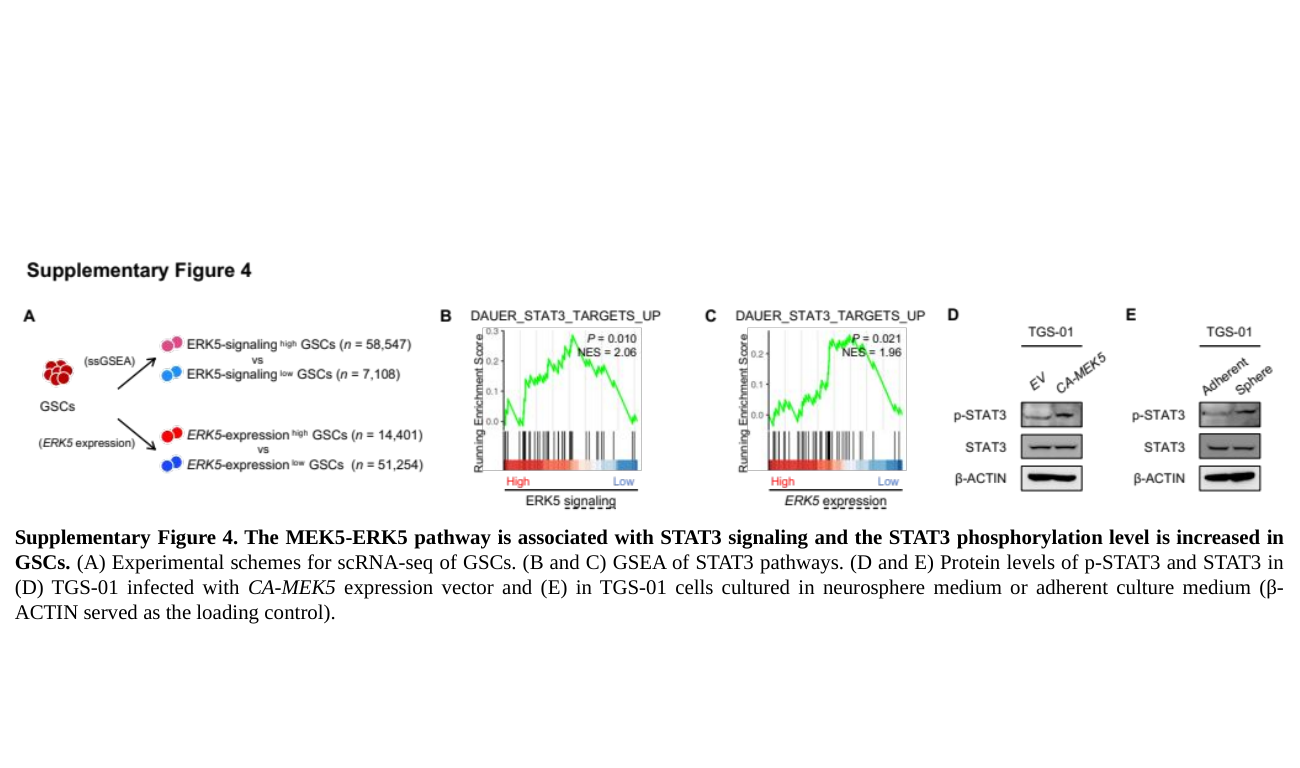

Supplementary Figure 4. The MEK5-ERK5 pathway is associated with STAT3 signaling and the STAT3 phosphorylation level is increased in GSCs. (A) Experimental schemes for scRNA-seq of GSCs. (B and C) GSEA of STAT3 pathways. (D and E) Protein levels of p-STAT3 and STAT3 in (D) TGS-01 infected with CA-MEK5 expression vector and (E) in TGS-01 cells cultured in neurosphere medium or adherent culture medium (β-ACTIN served as the loading control).
