# Supplementary material for: MEK5-ERK5 Axis Promotes Self-renewal and Tumorigenicity of Glioma Stem Cells
Source: Cancer Res Commun. 2023 Jan 30;3(1):148–59. doi: 10.1158/2767-9764.CRC-22-0243 (PMC10035453; doi:10.1158/2767-9764.CRC-22-0243)
Supplement: Figure S5 [file crc-22-0243-s06.pptx]

## Slide 1
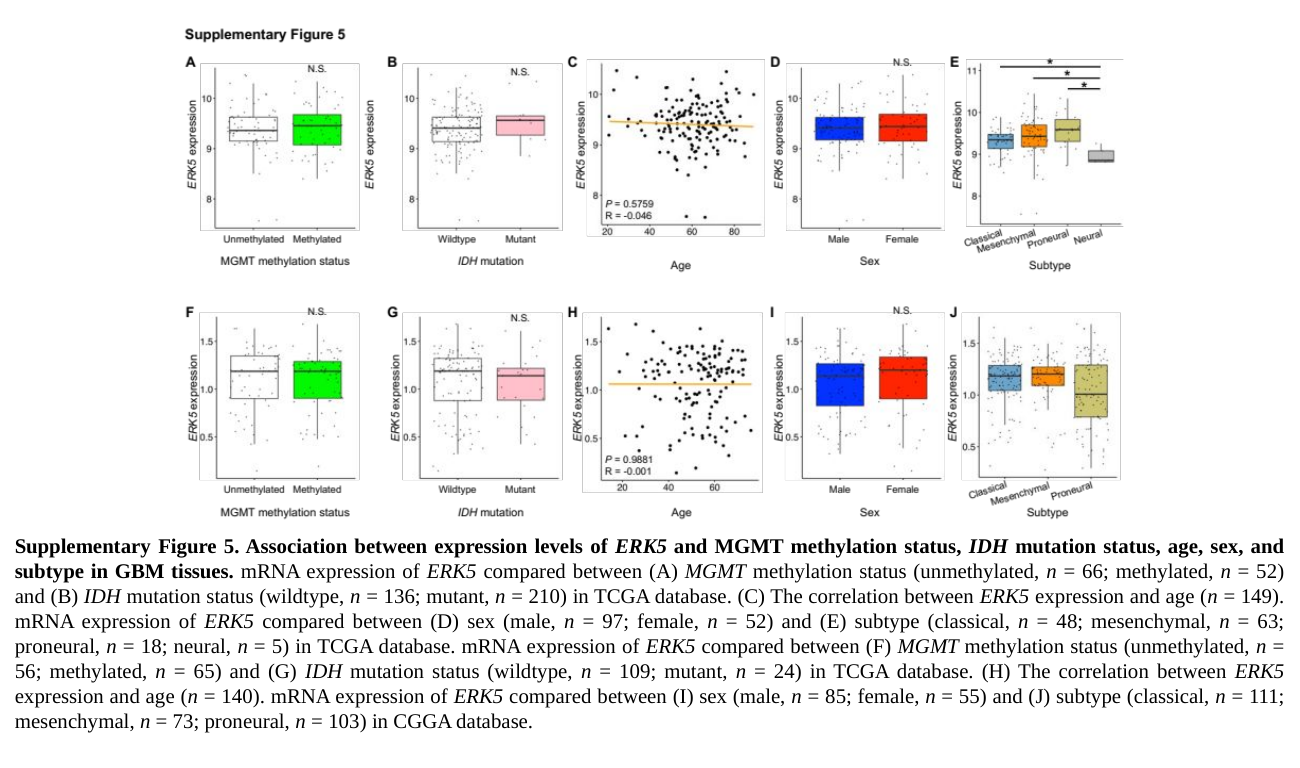

Supplementary Figure 5. Association between expression levels of ERK5 and MGMT methylation status, IDH mutation status, age, sex, and subtype in GBM tissues. mRNA expression of ERK5 compared between (A) MGMT methylation status (unmethylated, n = 66; methylated, n = 52) and (B) IDH mutation status (wildtype, n = 136; mutant, n = 210) in TCGA database. (C) The correlation between ERK5 expression and age (n = 149). mRNA expression of ERK5 compared between (D) sex (male, n = 97; female, n = 52) and (E) subtype (classical, n = 48; mesenchymal, n = 63; proneural, n = 18; neural, n = 5) in TCGA database. mRNA expression of ERK5 compared between (F) MGMT methylation status (unmethylated, n = 56; methylated, n = 65) and (G) IDH mutation status (wildtype, n = 109; mutant, n = 24) in TCGA database. (H) The correlation between ERK5 expression and age (n = 140). mRNA expression of ERK5 compared between (I) sex (male, n = 85; female, n = 55) and (J) subtype (classical, n = 111; mesenchymal, n = 73; proneural, n = 103) in CGGA database.
