# Supplementary material for: MEK5-ERK5 Axis Promotes Self-renewal and Tumorigenicity of Glioma Stem Cells
Source: Cancer Res Commun. 2023 Jan 30;3(1):148–59. doi: 10.1158/2767-9764.CRC-22-0243 (PMC10035453; doi:10.1158/2767-9764.CRC-22-0243)
Supplement: Figure S6 [file crc-22-0243-s07.pptx]

## Slide 1
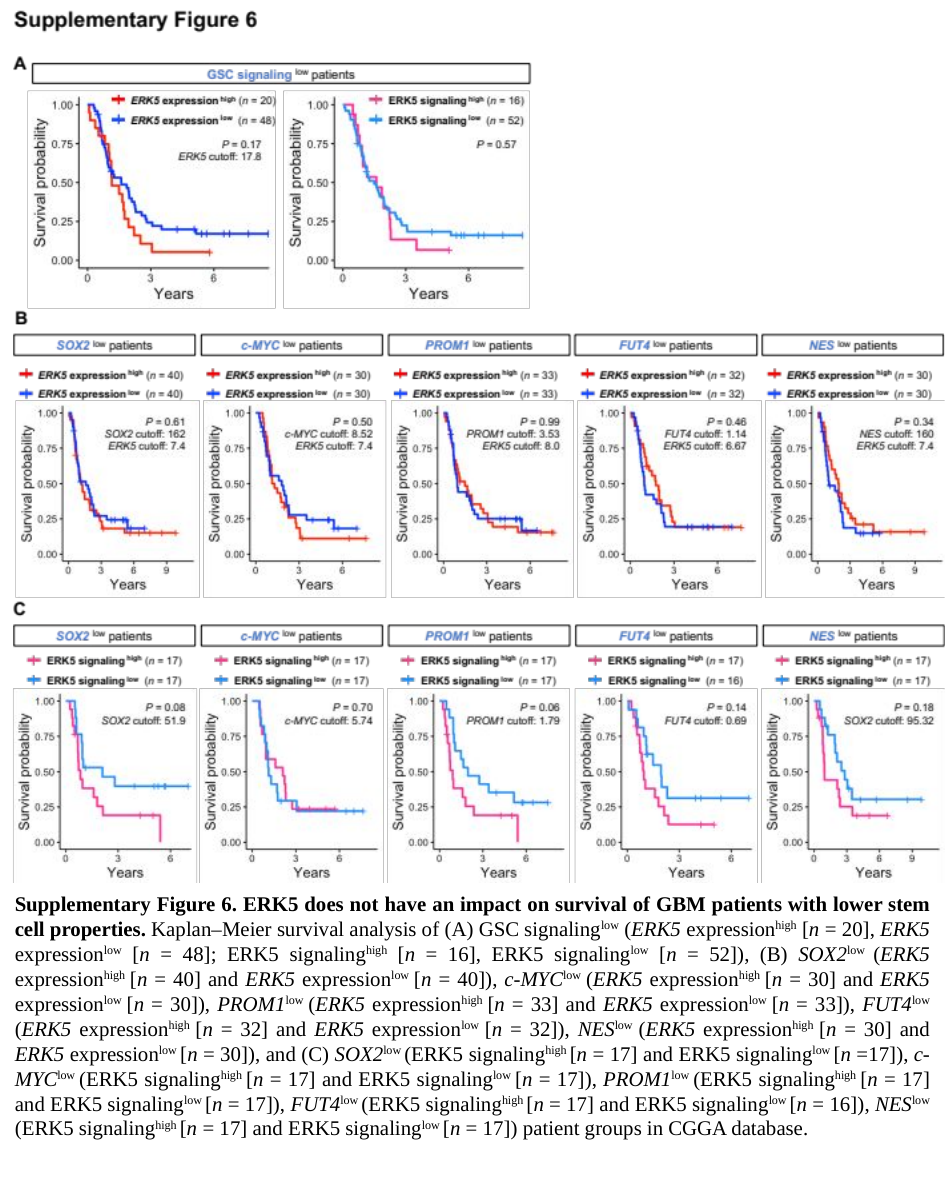

Supplementary Figure 6. ERK5 does not have an impact on survival of GBM patients with lower stem cell properties. Kaplan–Meier survival analysis of (A) GSC signalinglow (ERK5 expressionhigh [n = 20], ERK5 expressionlow [n = 48]; ERK5 signalinghigh [n = 16], ERK5 signalinglow [n = 52]), (B) SOX2low (ERK5 expressionhigh [n = 40] and ERK5 expressionlow [n = 40]), c-MYClow (ERK5 expressionhigh [n = 30] and ERK5 expressionlow [n = 30]), PROM1low (ERK5 expressionhigh [n = 33] and ERK5 expressionlow [n = 33]), FUT4low (ERK5 expressionhigh [n = 32] and ERK5 expressionlow [n = 32]), NESlow (ERK5 expressionhigh [n = 30] and ERK5 expressionlow [n = 30]), and (C) SOX2low (ERK5 signalinghigh [n = 17] and ERK5 signalinglow [n =17]), c-MYClow (ERK5 signalinghigh [n = 17] and ERK5 signalinglow [n = 17]), PROM1low (ERK5 signalinghigh [n = 17] and ERK5 signalinglow [n = 17]), FUT4low (ERK5 signalinghigh [n = 17] and ERK5 signalinglow [n = 16]), NESlow (ERK5 signalinghigh [n = 17] and ERK5 signalinglow [n = 17]) patient groups in CGGA database.
