# Supplementary material for: MEK5-ERK5 Axis Promotes Self-renewal and Tumorigenicity of Glioma Stem Cells
Source: Cancer Res Commun. 2023 Jan 30;3(1):148–59. doi: 10.1158/2767-9764.CRC-22-0243 (PMC10035453; doi:10.1158/2767-9764.CRC-22-0243)
Supplement: Figure S7 [file crc-22-0243-s08.pptx]

## Slide 1
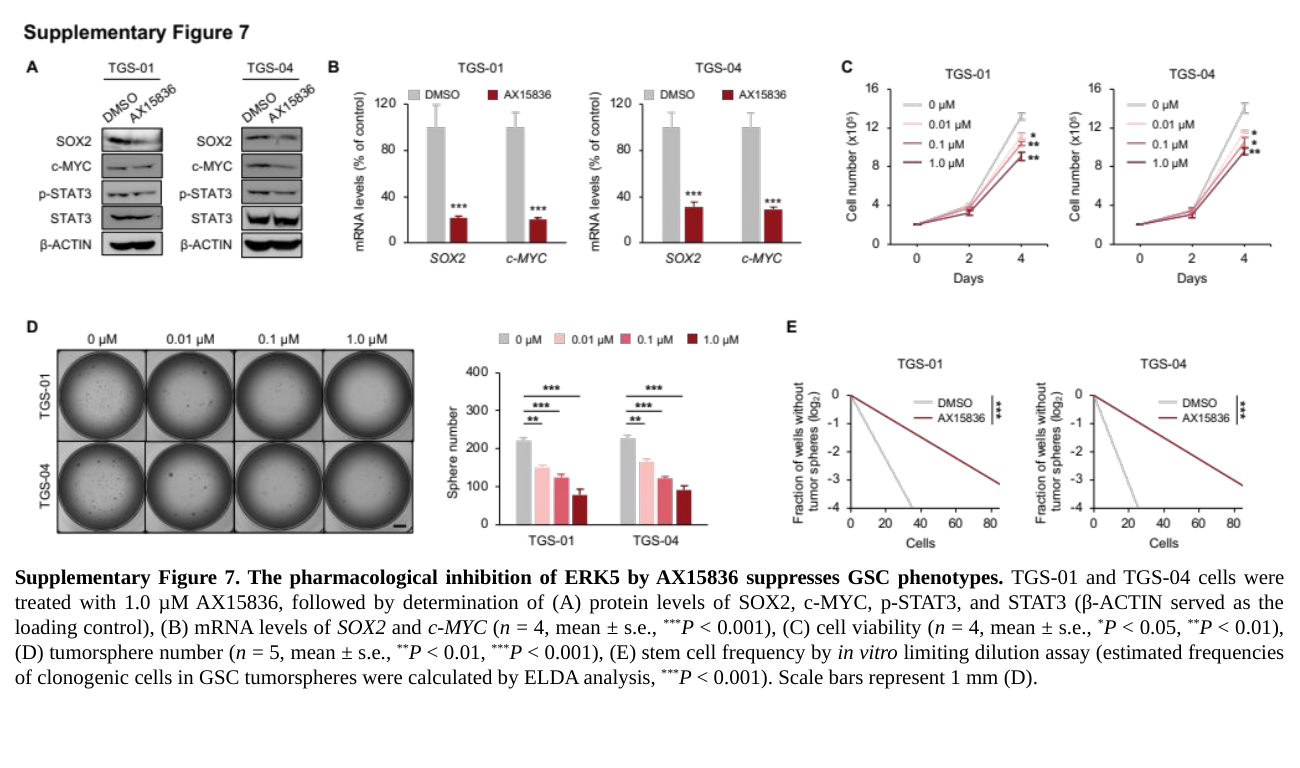

Supplementary Figure 7. The pharmacological inhibition of ERK5 by AX15836 suppresses GSC phenotypes. TGS-01 and TGS-04 cells were treated with 1.0 µM AX15836, followed by determination of (A) protein levels of SOX2, c-MYC, p-STAT3, and STAT3 (β-ACTIN served as the loading control), (B) mRNA levels of SOX2 and c-MYC (n = 4, mean ± s.e., ***P < 0.001), (C) cell viability (n = 4, mean ± s.e., *P < 0.05, **P < 0.01), (D) tumorsphere number (n = 5, mean ± s.e., **P < 0.01, ***P < 0.001), (E) stem cell frequency by in vitro limiting dilution assay (estimated frequencies of clonogenic cells in GSC tumorspheres were calculated by ELDA analysis, ***P < 0.001). Scale bars represent 1 mm (D).
